# Gene-based microsatellite development for mapping and phylogeny studies in eggplant

**DOI:** 10.1186/1471-2164-9-357

**Published:** 2008-07-30

**Authors:** Anikò Stàgel, Ezio Portis, Laura Toppino, Giuseppe Leonardo Rotino, Sergio Lanteri

**Affiliations:** 1 Di.Va.P.R.A. Plant Genetics and Breeding, University of Torino, Via L. da Vinci 44, 10095, Grugliasco (Torino), Italy; 2 CRA-ORL Research Unit for Vegetable Crops, Via Paullese 28, 26836 Montanaso Lombardo, LO, Italy

## Abstract

**Background:**

Eggplant (*Solanum melongena *L.) is a member of the *Solanaceae *family. In spite of its widespread cultivation and nutritional and economic importance, its genome has not as yet been extensively investigated. Few analyses have been carried out to determine the genetic diversity of eggplant at the DNA level, and linkage relationships have not been well characterised. As for the other *Solanaceae *crop species (potato, tomato and pepper), the level of intra-specific polymorphism appears to be rather limited, and so it is important that an effort is made to develop more informative DNA markers to make progress in understanding the genetics of eggplant and to advance its breeding. The aim of the present work was to develop a set of functional microsatellite (SSR) markers, via an *in silico *analysis of publicly available DNA sequence.

**Results:**

From >3,300 genic DNA sequences, 50 SSR-containing candidates suitable for primer design were recovered. Of these, 39 were functional, and were then applied to a panel of 44 accessions, of which 38 were cultivated eggplant varieties, and six were from related *Solanum *species. The usefulness of the SSR assays for diversity analysis and taxonomic discrimination was demonstrated by constructing a phylogeny based on SSR polymorphisms, and by the demonstration that most were also functional when tested with template from tomato, pepper and potato. As a results of BLASTN analyses, several eggplant SSRs were found to have homologous counterparts in the phylogenetically related species, which carry microsatellite motifs in the same position.

**Conclusion:**

The set of eggplant EST-SSR markers was informative for phylogenetic analysis and genetic mapping. Since EST-SSRs lie within expressed sequence, they have the potential to serve as perfect markers for genes determining variation in phenotype. Their high level of transferability to other *Solanaceae *species can be used to provide anchoring points for the integration of genetic maps across species.

## Background

The eggplant (*Solanum melongena *L.), also known as aubergine or brinjal, belongs to the *Solanaceae*, but unlike most of the solanaceous crop species, it is endemic to the Old, not the New World. Its progenitor is presumed to have been the African species *S. incanum *[1], but its centre of domestication and genetic diversity lies in the Indo-Burma region, where it has been grown for at least 1,500 years [2]. Despite its economic and nutritional importance, its genome has been little studied, in contrast to those of the other cultivated solanaceous crops tomato, potato and pepper, in which high density genetic linkage maps have been established [3-6]. The literature contains only a few reports describing RAPD [7], AFLP [8,9] and SSR [10,11] genotyping, a genetic map constructed with AFLP and RAPD markers [12] and a comparative genetic map, based on tomato sequences [13].

Microsatellites (SSRs) are short tandem repeats of simple (1–6 nt) motifs, and their value for genetic analysis lies in their multi-allelism, codominant inheritance, relative abundance, genome coverage and suitability for high-throughput PCR-based platforms [14]. It was long assumed that SSRs were primarily associated with non-coding DNA, but it has now become clear that they are also abundant in the single and low-copy fraction of the genome [15,16]. These latter SSRs are commonly referred to as "genic SSRs" or "EST-SSRs" and are present in 1 to 5% of the expressed plant DNA sequence deposited in public databases. With the increasing volume of publicly available unigene and cDNA sequences emerging from large-scale EST sequencing projects, the conventional need to generate enriched genomic libraries and to perform the necessary sequencing can now be largely bypassed [17]. Genic SSRs tend to be more readily transferable between (related) species or genera than genomic ones, since coding sequence is better conserved than non-coding sequence; however, they do tend to be less informative than conventional SSRs, particularly in the context of related genotypes [18,19]. On the other hand, they provide a powerful means to link the genetic maps of related species, and since many of them are located within genes of known or at least putative function, any allelic variation present can be exploited to generate perfect markers [20].

We present here our progress in the development and preliminary characterization of a set of eggplant SSR markers, derived from public database sequence, along with an evaluation of their experimental and *in silico *transferability among other solanaceous species.

## Results and discussion

### SSR motif frequency and distribution

At the time surveyed, the *Solanaceae *Genomics Network database (SGN; ) contained 3,181 eggplant ESTs, ordered into 1,841 unigenes (617 contigs and 1,224 singlets). An additional 176 sequences were retrieved from the EMBL sequence database . The non-redundant sequence pool contained 1,864 sequences representing 743,527 bp of genomic sequence. Within these, 64 contained one or more SSR (70 in total, including 20 mono-, 11 di-, 36 tri-, one tetra- and two hexanucleotide motifs). One sequence contained three SSRs, while ten SSRs were of the compound type (SSR containing stretches of two or more different repeats). The mean separation between two SSRs was ~10.6 kb, equivalent to one SSR per 29 sequences. This distance is somewhat greater than that estimated for several monocotyledenous [15,21] and dicotyledenous [22] species, perhaps because of the greater stringency of the criteria and the lesser size of the sequence dataset.

The properties of the 70 SSR loci identified are summarised in Table 1, classified on the basis of repeat motif and the number of repeat units. Trinucleotides were the most frequent (51.4%), followed by mono- (28.6%) and dinucleotides (15.7%). Tetra- and hexanucleotides were rare. Although trinucleotide motifs are less frequent in genomic libraries, they represent the most common class in expressed sequence [18,23,24], since variation in repeat number does not normally affect downstream peptide sequence, unlike mono-, di-, or tetra-nucleotide motifs, which generate frameshift mutations and therefore are more likely to be selected against [25]. All ten possible trinucleotide motifs were recovered, with AAG/CTT the most frequent (30.6%), as has been seen in other *Solanaceae *species [26,27] and more generally within plant sequence databases [16,28]. CCG/CGG and AGG/CCT are the most common monocotyledonous EST-SSR motifs [18,24,29] and were under-represented in dicotyledonous species as well as in the present dataset. Kantety et al. [30] have observed that AG/CT predominates among the dinucleotide motifs, presumably reflecting the high frequency of Ala (AGA) and Leu (GAG) (respectively, 8% and 10%) in polypeptides [31]. These motifs represented 45.5% of the eggplant dinucleotide SSRs. The second most abundant motif (36.4%) was AT/AT, which is also well represented among plant EST sequences [32,33]. Most of the mononucleotide repeats (19/20) were A/T.

**Table 1:** Occurrence of non-redundant SSRs in a set of 3,357 *Solanum melongena *sequences.

**SSR motif**	**Number of repeats**												**Total**
		
	**4**	**5**	**6**	**7**	**8**	**9**	**10**	**11**	**12**	**13**	**14**	**>15**	
A/T	-	-	-	-	-	-	-	-	2	3	2	12	19
C/G	-	-	-	-	-	-	-	-	1	-	-	-	1
AC/GT	-	-	-	1	1	-	-	-	-	-	-	-	2
AG/CT	-	-	-	3	1	-	-	-	-	-	-	-	4
AT/AT	-	-	-	-	2	2	-	-	1	-	-	-	5
AAC/GTT	-	1	-	-	-	-	-	-	-	-	-	-	1
ACG/CTG	-	2	1	-	-	-	-	-	-	-	-	-	3
AAG/CTT	-	10	-	-	-	1	-	-	-	-	-	-	11
AAT/ATT	-	1	-	-	-	-	-	-	-	-	-	3	4
ACC/GGT	-	2	-	-	-	-	-	-	-	-	-	-	2
ACT/ATG	-	3	-	1	-	-	-	-	-	-	-	-	3
AGC/CGT	-	2	-	-	1	-	-	-	-	-	-	-	4
AGG/CCT	-	1	-	-	-	-	-	-	-	-	-	-	1
AGT/ATC	-	5	-	1	-	-	-	-	-	-	-	-	6
CCG/CGG	-	1	-	-	-	-	-	-	-	-	-	-	1
AAAT/ATTT	-	-	1	-	-	-	-	-	-	-	-	-	1
AACCAG/CTTGGT	1	-	-	-	-	-	-	-	-	-	-	-	1
ACCAGC/CGTGGT	-	-	1	-	-	-	-	-	-	-	-	-	1

N	-	-	-	-	-	-	-	-	3	3	2	12	20
NN	-	-	-	4	4	2	-	-	1	-	-	-	11
NNN	-	28	1	2	1		-	-	-	-	-	3	36
NNNN	-	1	-	-	-	-	-	-	-	-	-	-	1
NNNNNN	1	-	1	-	-	-	-	-	-	-	-	-	2

**Total**													**70**

The total length of the 64 microsatellite containing sequence reached the 31,909 bp. Of this 16,862 bp represented untranslated (UTR) – and 15.047 bp represented protein-coding regions. SSRs were non-randomly distributed among coding regions and UTRs. All of the mononucleotide and majority of the dinucleotide repeats (91%) were associated with UTRs. Mononucleotide repeats were evenly distributed among 5' and 3' UTRs while dimeric ones preferentially associated with 5'UTRs. Triplet repeats were significantly over-represented in coding region (75%) and among non-coding regions showed more than 3 folds greater frequency in 5'UTRs. Such dominance of trimeric over other SSRs in coding regions can be explained by non-perturbation of the reading frame.

### SSR assays and their informativeness

Of the 64 sequences containing one or more SSR, 50 (78%) were amenable to primer design. The markers targeted by EEMS01 to EEMS50 comprised 15 mono-, five di-, 24 tri- and two hexanucleotide simple repeats, together with two di- and two trinucleotide compound loci. The remaining sequences contained either too little flanking sequence, or the sequences themselves were refractory for primer design. Thus, primers amplifying non-redundant loci were designed from about 1.4% of the initial number of database sequences, a success rate comparable to that experienced in other species [23,26,27]. Amplicons were generated from genomic DNA template from 39 (78.0%) of the 50 loci. Failure to amplify can be due to a variety of causes, including the positioning of primers across a splicing site, or to a chimeric origin of the cDNA clones. In all, 31 (79.5%) of the 39 assays were informative across the whole genotype panel (Table 2), but only 11 (28.2%) were informative among the sample of cultivated eggplant. The majority of the trinucleotide-containing SSRs were informative between species, but few generated any polymorphism among the cultivated set, while the dinucleotide SSRs identified both inter- and intra-specific polymorphism. Similar results have been reported for eggplant by Nunome et al. [10,11] who described that 57% of trinucleotide SSRs were informative at inter-, but only 14% at intraspecific level, while, for the dinucleotide SSRs, the respective frequencies were 78% and 70%. The repeat type, primer sequence and PIC (polymorphism information content) of the successful markers are given in Table 3.

**Table 2:** *Solanum melongena *(Sm) genotypes and *Solanum *related wild species (Sr) assayed (shape and skin colour are indicated in bracket).

**Species**	**Genotypes**	**Use^1^**	**codes**
*S. melongena*	Angió3 (Long purple)	BL	Sm-1
	Angió5 (Long green)	BL	Sm-2
	ANK1 (Oval white purple striped)	BL	Sm-3
	ANK2 (Oval white purple striped)	BL	Sm-4
	Anominori (Long purple)	CV	Sm-5
	Baffa (Oval purple)	CV	Sm-6
	Bianca stirata verde (Small white green striped)	BL	Sm-7
	Buia (Oblong purple-black)	BL	Sm-8
	Cannellina Sarnese (Small long purple)	CV	Sm-9
	CN-2/Qiyeqie (Round purple)	CV	Sm-10
	Daizaburou (long purple)	RT	Sm-11
	Diataro (round purple)	RT	Sm-12
	Dourga (Long white)	CV	Sm-13
	DR2 (Long dark purple)	BL	Sm-14
	Gadilak F1 (Long purple)	CV	Sm-15
	GIC (Oblongl purple-black)	BL	Sm-16
	Hympulse (Long purple-black)	CV	Sm-17
	JM (Small elongate light purple)	CV	Sm-18
	Lunga violetta (Long purple)	CV	Sm-19
	Lunga violetta napoletana (Long light purple)	CV	Sm-20
	Maya (Oval purple)	CV	Sm-21
	Mirabelle (Long purple-black)	CV	Sm-22
	Mostruosa di New York (Oval purple)	CV	Sm-23
	Ovale piccola bianca/egg (Small oval white)	BL	Sm-24
	Palermitana (Oval light purple)	CV	Sm-25
	Pusa purple cluster (Small elongate purple)	CV	Sm-26
	Pusa purple long (Long purple)	CV	Sm-27
	Sita 07 (Oval light purple)	BL	Sm-28
	SM19/14 (Long purple)	BL	Sm-29
	Tanindo Subur (Long light purple)	CV	Sm-30
	Tian long (Long purple)	CV	Sm-31
	Tina (Long dark purple)	BL	Sm-32
	Tunisina Baharia (Oval light purple)	CV	Sm-33
	Violetta di Firenze (Oval light purple)	CV	Sm-34
	Violetta lunga semiorto (Long dark purple)	CV	Sm-35
	Zihzung F1 (Long purple)	CV	Sm-36
	305 E40 (Long purple-black)	BL	Sm-37
	67-3 (Oval light purple)	BL	Sm-38
*S. viarum*	Japan		Sr-1
*S. sodomaeum*	Italy		Sr-2
*S. sisymbrifolium*	USA		Sr-3
*S. torvum*	Indonesia		Sr-4
*S. aethiopicum*	France		Sr-5
*S. integrifolium*	Japan		Sr-6

^1^BL: breeding line; CV: cultivated variety; RT: rootstock.

**Table 3:** Allelic variation in 39 SSR loci.

**Code**	**Repeat**	**FORWARD PRIMER (5'-3')**	**REVERSE PRIMER(5'-3')**	**Expected size of alleles (bp)**	**Allele size range (bp)**	**Nr. of alleles^1^**		**PIC^2^**		**SSR position^3^**
								
						**NA1**	**NA2**	**PICm**	**PICs**	
EEMS06	(T)14	TCATGCGAAGATTAATTAAATGTGA	GAGTGGATGATCAAGAATGGC	265	268–274	1	4	0	0,174	3' UTR
EEMS07	(T)13	CCATGCCAGAATGGAAACTT	AACGAAAACACGATCAACCC	247	250–260	1	4	0	0,209	3' UTR
EEMS10	(A)20	TCAAGCAGAACGAAGATGGA	GTAGGGGACGTGGATTCAGA	282	266–290	1	4	0	0,174	3' UTR
EEMS12	(A)16	CGGGCAACTCTTCACATTTT	ATTGGTTTGCTATCGAATTTCT	158	146–150	2	3	0,097	0,273	5' UTR
EEMS13	(A)14	TGAGATACGCGTACAATGACTTC	GGGGTTTTGCTGCTGTTATC	140	140	1	1	0	0	5' UTR
EEMS14	(A)13	GGAATGGACCAAACCCCTAA	AGAGCTTCGTTGCTTGGTGT	277	270–276	1	3	0	0,088	5' UTR
EEMS15	(C)12	GGGACAAATCTGACCTTTGG	CTGGTGGCAAATTCTTCGAT	292	270–294	5	7	0,645	0,755	3' UTR
EEMS16	(AC)7	CAATTTTTCGGTTCACTAATCAAG	CTTCAAGGAAAAAGGAGGCC	132	135–141	3	4	0,135	0,260	3' UTR
EEMS17	(CA)8	TGACATGTAGCTGGGCAGAG	TGGAGTGTGCATCCCAAATA	197	195–197	2	2	0,492	0,499	3' UTR
EEMS18	(AG)7	GGAGAAACTGAAAAATTTGTAGAGAG	GAGGAGTTTCCGACATGAGC	187	183–187	1	3	0	0,126	CDS
EEMS19	(AT)9	GGCATGACAAAATCATACAAACA	TGTTGGTTAAGTCCATGGGAA	173	165–177	1	3	0	0,135	3' UTR
EEMS20	(AT)8	AACATCAGCCAGGGTGTTTC	TGCTGAAAATTACAAGCCAAA	215	221–227	2	4	0,049	0,278	3' UTR
EEMS21	(AGA)5	TGATGTTGAACCGACACAAGA	CGTCTTCATCTTCCTCCTCG	131	122–140	1	3	0	0,126	CDS
EEMS22	(AAG)5	GAAGGACGTTGGTCCTGGTA	CTGTTCATTATCCCCATCGC	162	165–168	1	2	0	0,085	CDS
EEMS23	(TTC)5	CACCAATTTCCCCCTTCTTT	CGGTTGGTAAAGAAAACCCA	144	145	1	1	0	0	CDS
EEMS24	(CTT)5	CACCTGTTTGAGCACCTTGA	CACCGAAGGCAGAGAAGAAG	221	217–220	1	2	0	0,043	CDS
EEMS25	(CTT)5	CCCATAGCTTTGCTCGAGAT	GCACCAAAGGCAGAGAAGAA	227	225–230	1	2	0	0,043	CDS
EEMS26	(CTT)5	GACACTCCCCTACTTCCACCT	CGCTTAGCAGAAGCCGATAA	260	355–360	1	2	0	0,087	CDS
EEMS28	(TAA)21	GACGATGACGACGACGATAA	TGGACTCACAACTCAGCCAG	219	180–236	7	9	0,665	0,714	3' UTR
EEMS29	(ATG)5	TCAGTCAACTGCATCACCAGA	ATTCCCATTATTGGCTGCTG	118	120	1	1	0	0	CDS
EEMS30	(TAC)5	TTTACATGACAGCACCAGGC	ATTTTATGGGAATGGGGTCC	191	189–195	1	2	0	0,087	3' UTR
EEMS31	(TGG)5	GAGAAGTTGGCTTCAGTGCC	TAAACTCAAGGGATGCTGGG	239	330–339	1	2	0	0,043	CDS
EEMS32	(TCA)5	TAAGGAGTCTGATGCCGCTT	GTAATGCTCCTCCACGGCTA	151	150	1	1	0	0	CDS
EEMS33	(TCA)5	CTATCTCCTTTTCCCCGACC	ATGAATAAGCTGCCACCACC	220	222	1	1	0	0	CDS
EEMS34	(TCA)5	GCTTGATTCCCCACAAAGAA	GTTTCATCGCCCTCATCATT	276	275–278	2	2	0,123	0,143	CDS
EEMS35	(TCA)5	ATGGCTTCTGATGGACCAAG	CACTTGATGAACGTGGATGG	230	232	1	1	0	0	CDS
EEMS36	(TGT)5	TCTATCATCCCCAGATCCCA	AAGGTCGCATGGACATTAGG	117	110–120	1	2	0	0,043	CDS
EEMS37	(TCC)5	CCCTTCCTACCCACACTTCA	GTTTTGCACCTTTCCATCGT	117	114–123	2	4	0,375	0,502	CDS
EEMS38	(CAC)5	TTCAATCGAACTTCGGAACC	ATGACGGTGGATCTCGCTAC	148	135–153	1	3	0	0,086	CDS
EEMS39	(CTG)5	GGAGAGATGGATGCCGAATA	TCTCGACCTTAGCCTGCATT	166	264–270	1	3	0	0,126	CDS
EEMS41	(GCA)5	ATTCTGCATTCATCGGAAGG	GGATTGCTTGTGGGAATATCA	260	700, 1600	1	1	0	0	CDS
EEMS42	(GCA)6	GCTCAGCAACCACAGTACCA	GTCCGGACTTCATCAGCATT	152	155–180	1	3	0	0,166	CDS
EEMS44	(GCC)5	CCTTCAAACCCTCTCCCTTC	GTGAAACGTGGTGGAGGTCT	216	215	1	1	0	0	CDS
EEMS45	(AGAACC)4	AGCGCTTGTCCAGGCTATAA	TTTCCACCATGAGCAAATGA	282	279–285	1	2	0	0,197	
EEMS46	(ACCAGC)6	ACCAAACGTGCATGAAACAA	GGAAATGTTGGTGGAATTGG	264	245–265	1	4	0	0,207	CDS
EEMS47	(GCT)5..(TTC)5	CGAACACATTCGCAAATCAC	GCATCACAAGGATGGAAAGG	246	250–253	1	2	0	0,162	CDS
EEMS48	(TAA)20(CGA)8	CAATGCAAACAATTATCATTTCG	TCGATGTTGTTGTCGTCGTT	213	223–241	7	9	0,641	0,677	3' UTR
EEMS49	(TA)12(GA)7	TGAAATTGATCAATACCTATAAATTTAG	GAAAGCCAGGATAGCATTCG	140	145–153	5	5	0,677	0,677	3' UTR
EEMS50	(TA)9(GA)8	AAATCCGGCCATTCTGTGTA	ACATCGTTCCGCCTCTATTG	224	218–226	2	4	0,229	0,377	5' UTR

^1 ^NA1: number of alleles detected among the 38 cultivated types, NA2: number of alleles detected among the full 44 genotype set; ^2 ^PICm: calculated among the 38 cultivated types, PICs: calculated among the full 44 genotype set; ^3^CDS: Coding sequence

Generally, amplicon size was in agreement with expectation, although EEMS 26, 31, 39 and 41 all amplified a product at least 100 bp larger than expected, presumably because the amplicon included an intron. EEMS12 produced an amplicon of smaller than expected length, perhaps because of the presence of a deletion within the genomic sequence, poor priming specificity amplifying a non-target member of a gene family, or because of minor sequence variation between the amplified copy and the consensus sequence [34]. A total of 116 alleles was amplified from the full genotype panel, with the number of alleles per locus varying between 1 and 9 (mean 3.1) (Table 3). The greatest variation in amplicon size (180–236 bp) was shown by EEMS28. Both the PIC among the 38 cultivated types (PIC_m_) and among the full 44 genotype set (PIC_s_) were calculated. PIC_m _ranged from 0.05 to 0.68 (mean 0.38 ± 0.12), while PIC_s _varied from 0.04 to 0.76 (mean 0.24 ± 0.09). The highest and lowest PIC_m _were produced by, respectively, EEMS49 and EEMS20, while EEMS15 had the highest, and EEMS24, 25, 31 and 36 shared the lowest PIC_s_. The correlation coefficient between PIC_m _and SSR length was 0.6 (*p *= 0.0001), in agreement with the general trend for long SSRs to be more informative than shorter ones [35]. Trinucleotide motif SSRs were less informative than the dinucleotide types (PICs of 0.16 and 0.26 respectively). The former are typically associated with a low level of variability [18,36]. The overall level of intraspecific polymorphism uncovered (28.2%) is typical [37-39], and compares poorly with the rate achievable by genomic SSR assays [37,40,41].

### Genetic diversity revealed by SSR markers

**Figure 1:**
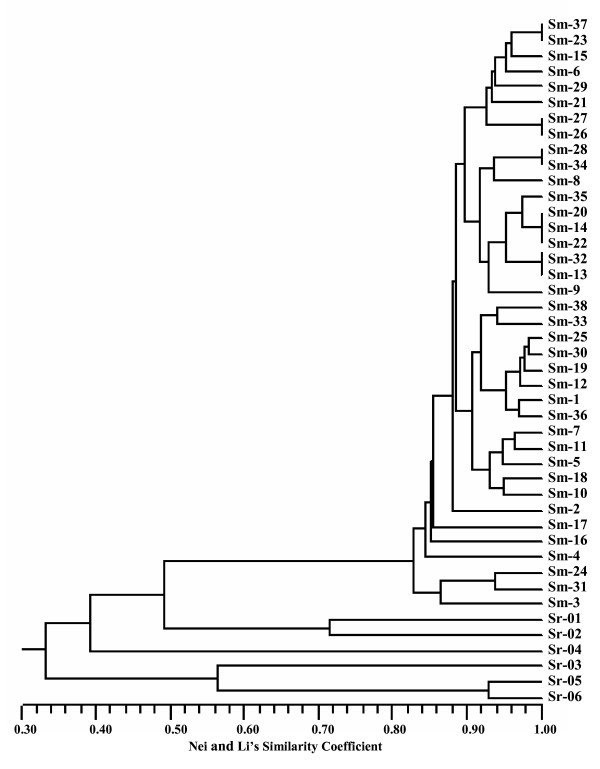
**UPGMA dendrogram**. Analysis of the 44 genotype set, based on 116 EST-SSR alleles. Sample codes are described in Table 2.

Thiel et al [24] have stressed the limitations surrounding the application of SSR markers for diversity studies, emphasising the possibility of homoplasy (identical allele sizes may not be identical by descent), and have pointed out that allele size differences can also be generated by indel events, as well as by variation in the SSR repeat number. However, the genetic relationships between the accessions of the full genotype panel as displayed by genetic similarity at the SSR level were in good agreement with prior taxonomic classification based on both genomic [9,11] and plastidial markers [42,43]. Thus the cultivated eggplants clustered with an average genetic similarity of 82% (Figure 1). Three pairs of cultivars ('Tina' and 'Dourga'; 'Sita 07' and 'Violetta di Firenze'; 'Mostruosa di New York' and '305 E40') and 'Mirabelle', 'DR2' and 'Lunga violetta napoletana' were identical to one another. The cluster closest to the cultivated group contained both *S. viarum *and *S. sodomaeum*, with a mean genetic differentiation of ~50% from the cultivated germplasm. The *S. torvum *accession was more distant (mean genetic similarity 39%). The third cluster contained the remaining species *S. sisymbrifolium, aethiopicum *and *integrifolium *which shared a mean genetic similarity of 56%.

The EEMS primers were also applied to amplify template from potato, tomato and pepper, which all belong to the *Solanaceae*. To minimise non-specific amplification, the same stringency level for PCR was applied as with eggplant template. About 54% (21 of the primer pairs) generated a detectable amplicon from at least one of the three species; ten of 21 amplified all three templates, seven amplified potato and tomato but not pepper DNA, two tomato and pepper but not potato, and one each amplified only from potato and tomato.

**Figure 2:**
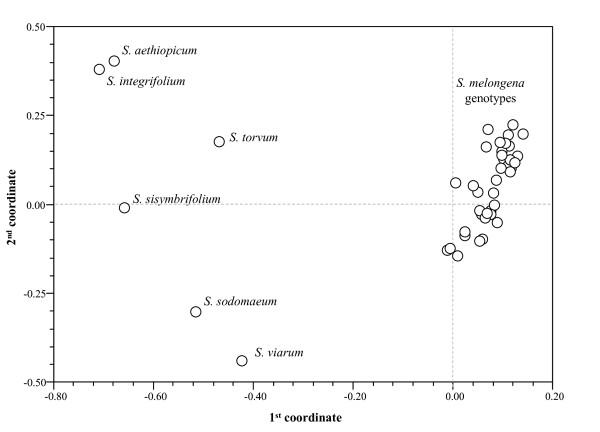
**Biplot of the Principal co-ordinates analysis**. Analysis based on microsatellite data depicting the genetic relationship among the 44 *Solanum *genotypes.

The principal co-ordinate analysis (PCO) analysis illustrates the genetic relationships between the members of the genotype panel (Figure 2). The first three principal co-ordinates accounted for ~54% of the overall genetic variation, with each in turn contributing 34.2%, 10.3% and 9.4%. The first co-ordinate distinguished the cultivated forms from the allied genotypes, while the second allowed the separation of each related eggplant genotypes.

### BLAST analyses

Of the 39 functional SSR markers, all but EEMS45 were developed from anonymous eggplant unigene sequences, 25 of which share significant homology to *Arabidopsis thaliana *proteins of unknown function. EEMS45 lies within a chloroplast phosphate transporter gene (Table 4). Using the source eggplant sequences as a BLASTN query (the target database has been described in the 'Method' section), 24 (61.5%) of the markers identified highly conserved orthologs, with a frequency negatively correlated with phylogenetic distance from eggplant [44]. EEMS15, EEMS21, EEMS24, EEMS39, and EEMS45 had homologous counterparts with known function. Sequences containing homologous microsatellite motifs in conserved positions were found in 15 potato, 10 tomato and 1 pepper orthologs (Table 4). Contrasting results are reported in literature on the transferability of microsatellite markers across members of the *Solanaceae *[26,45,46]. The high level of transferability between the seven *Solanum *spp. mirrors the experience in other groups of plants [47]; furthermore we detect a low level of intraspecific polymorphism which seems to confirm the conclusion that EST-SSRs are highly conserved across species [48].

**Table 4:** Homology relationships of the EEMS markers.

**Marker**	**SGN Unigene ID**	**Homologous *Arabidopsis *peptide**	**Homologous ESTs in tomato, potato or pepper (GenBank ID)**	**Annotation**	**e-value**	**SSR in the same position**
EEMS06	U206099		DN587316	47382.1 Late Blight-Challenged Tubers Solanum tuberosum cDNA clone 47382	e-100	
			AW029731	EST272986 tomato callus, TAMU Lycopersicon esculentum cDNA clone cLEC28K19	7e-80	
EEMS07	U206473	At3g55570.1	CV506255	72934.1 Mixed Floral Solanum tuberosum cDNA clone 72934	2e-77	
			CA525885	KS12063A07 KS12 Capsicum annuum cDNA	1e-75	
			AK224899	Solanum lycopersicum cDNA, clone: FC25DG07, HTC in fruit	6e-65	
EEMS10	U206024		CK274806	EST720884 potato abiotic stress cDNA library Solanum tuberosum cDNA clone POADJ52	e-164	+
EEMS14	U205878	AtCg00070.1	DQ347958	Solanum bulbocastanum cultivar PT29 chloroplast, complete genome	0.0	
			DQ347959	Lycopersicon esculentum cultivar LA3023 chloroplast, complete genome	0.0	+
			ER831875	PPTC658TF Solanum tuberosum RHPOTKEY BAC ends Solanum tuberosum genomic clone RHPOTKEY138_J19, genomic survey sequence	0.0	+
EEMS15	U207285	At1g15820.1	BI435095	EST537856 P. infestans-challenged potato leaf, compatible reaction Solanum tuberosum cDNA clone PPCBZ49	e-139	
			M32605	Tomato chlorophyll a-binding protein (Cab10A) gene	e-124	
EEMS17	U206974	At5g53360.1	AA824717	CT008.SK Tomato Leaf cDNA from cv. VFNT cherry Lycopersicon esculentum cDNA clone CT008	1e-71	+
			DN587261	47295.1 Late Blight-Challenged Tubers Solanum tuberosum cDNA clone 47295	3e-60	
EEMS18	U205890	At1g08200.1	BI932492	EST552381 tomato flower, 8 mm to preanthesis buds Lycopersicon esculentum cDNA clone cTOC23G14	0.0	
EEMS20	U206004	At5g52990.1				
EEMS21	U207374	At3g56860.3	DQ284462	Solanum tuberosum clone 072A05 RNA-binding protein AKIP1-like mRNA	e-148	
EEMS22	U206874	At1g07790.1	AC204499	Solanum tuberosum chromosome 6 clone RHPOTKEY069B12	e-129	+
			AI778436	EST259315 tomato susceptible, Cornell Lycopersicon esculentum cDNA clone cLES5G8	e-127	+
			CA524430	KS12037D12 KS12 Capsicum annuum cDNA, mRNA sequence	e-111	
EEMS23	U207287	At2g25080.1	BQ113411	EST598987 mixed potato tissues Solanum tuberosum cDNA clone STMCN43	e-126	+
			ES890426	LET011F7_2005-09-27_1/LET011F7_A12_1 Solanum lycopersicum trichomes	e-114	+
EEMS24	U205612	At5g59910.1	DQ268853	Solanum tuberosum clone 167E08 histone H2B-like protein mRNA	0.0	+
EEMS25	U205886	At5g59910.1	BG643224	EST511418 tomato shoot/meristem Lycopersicon esculentum cDNA clone cTOF26P12 5' sequence	e-102	+
			CV501903	66441.1 Mixed Floral Solanum tuberosum cDNA clone 66441	3e-86	+
EEMS26	U205659	At5g05270.2				
EEMS28	U205759	At5g20950.2	CK262774	EST708852 potato abiotic stress cDNA library Solanum tuberosum cDNA clone POABI35	e-143	+
EEMS29	U206036	At5g38050.1	BP877982	Solanum lycopersicum cDNA, clone: FA10BF05, 5' end, expressed in maturing fruit	7e-98	+
EEMS30	U206347		BQ508532	EST615947 Generation of a set of potato cDNA clones for microarray analyses mixed potato tissues Solanum tuberosum cDNA clone STMGW83	e-112	+
EEMS31	U206031	At2g27710.2	CV469914	42678.1 Common Scab-Challenged Tubers Solanum tuberosum cDNA clone 42678	0.0	+
			DB680885	Solanum lycopersicum cDNA, clone: LEFL1008CF04, 5' end, expressed in leaf	0.0	+
EEMS32	U207015	At4g19430.1				
EEMS35	U205935	At1g62045.1	CK861590	32687 In vitro Root Solanum tuberosum cDNA	e-105	+
			AC215407	Solanum lycopersicum Tomato chromosome 2, C02HBa0167J21, complete sequence.	8e-89	
EEMS37	U206679	At5g64280.1				
EEMS38	U205635	At2g21660.1	CK277760	EST723838 potato abiotic stress cDNA library Solanum tuberosum cDNA clone POAE172	e-113	+
EEMS39	U206514	At5g51120.1	AI482858	EST242181 tomato shoot, Cornell Lycopersicon esculentum cDNA clone cLEB3D24 similar to RNA binding protein	e-108	+
EEMS41	U205902	At3g04940.1				
EEMS42	U206785	At1g11650.1	CK271457	EST717535 potato abiotic stress cDNA library Solanum tuberosum cDNA clone POACZ28	e-132	+
			AI781607	EST262486 tomato susceptible, Cornell Lycopersicon esculentum cDNA clone cLES16F7	e-125	+
EEMS44	U205885	At2g43090.1	CK276247	EST722325 potato abiotic stress cDNA library Solanum tuberosum cDNA clone POADS32 5' end, mRNA sequence.	e-148	+
EEMS45	U94558		BQ047601	P. infestans-challenged potato leaf, incompatible reaction Solanum tuberosum cDNA clone BPLI18O9	0.0	+
			EF094557	Capsicum frutescens chloroplast phosphate transporter (Pht2;1)	0.0	+
			BE433007	EST399536 tomato breaker fruit, TIGR Lycopersicon esculentum cDNA clone cLEG11H11	2e-98	+
EEMS48	U205759	At5g20950.2				
EEMS49	U206896		EI386298	POTCQ36TF Solanum tuberosum RHPOTKEY BAC ends Solanu tuberosum genomic clone RHPOTKEY025_F23, genomic survey sequence.	1e-37	
EEMS50	U205674	At2g43360.1	BF187639	EST443926 potato stolon, Cornell University Solanum tuberosum cDNA clone cSTA41B6	0.0	+
			BI935563	EST555452 tomato flower, anthesis Lycopersicon esculentum cDNA clone cTOD23C22	e-167	

## Conclusion

In eggplant, as in pepper and tomato [3,49,50], the level of intraspecific DNA marker polymorphism is rather limited. Nunome et al [11] constructed a genetic map in eggplant based on RAPD and AFLP markers, but only 8.3% of the RAPD primers were informative, and even the AFLP primer combinations were only able to deliver a mean of 2.4 polymorphisms each. We have shown that an *in silico *analysis of the albeit limited quantity of publicly available eggplant DNA sequence has enabled the development of a set of functional SSR markers. Because these sequences are derived from the expressed portion of the genome, they are relevant for assaying functional diversity in populations or germplasm collections. Most of the EEMS SSRs are readily transferable to related species, and so can be exploited as anchor markers for comparative mapping and evolutionary studies.

## Methods

### Mining of SSR-containing sequences and primer design

In all, 3,357 eggplant sequences were retrieved from the SGN and EMBL nucleotide databases, using the Sequence Retrieval System (SRS6, ). A stand-alone nucleotide database was built for local BLAST2 searches [51]. PolyA and polyT tracts were removed, by applying the criterion that no 50 bp window contain a run of ten A's or ten T's. ClustalW [52] alignment was used to eliminate redundancy, by setting the following two criteria: (i) where a cluster contained two or more identical sequences, the longest was retained, and (ii) where the members of a cluster fell into recognisable sub-groups, only one member of each sub-group was retained. Sequences composed entirely of SSR motif (i.e., lacking any flanking sequence) were discarded, since their uniqueness could not be established, and in any case, primer design is not possible. SSR-containing sequences were identified using MISA software [24], a Perl script which allows both perfect and compound SSRs to be detected. A sequence was considered an SSR where a motif was repeated at least 12 times (1 nt motif), seven times (2 nt) or five times (3–6 nt), allowing for only one mismatch. For compound repeats, the maximum default interruption (spacer) length was set at 100 bp.

Primer pairs were designed from the flanking sequences, using PRIMER3 software [53] in batch mode via the *p3_in.pl *and *p3_out.pl *Perl5 scripts within the MISA package. The target amplicon size was set as 100–300 bp, the optimal annealing temperature as 60°C, and the optimal primer length as 20 bp. The resulting markers were each assigned the prefix EEMS (EST Eggplant MicroSatellite). Local BLASTN analyses were carried out using all EEMS sequences as queries. The target database contained 1,524,584 entries derived from a variety of solanaceous species, retrieved from the EMBL sequence database (Release 93)

### Plant material, DNA extraction and PCR

EEMS informativeness was evaluated using a panel of 44 accessions, made up from 38 cultivated eggplant varieties, breeding lines and rootstocks, and six related wild *Solanum *species (Table 2). Cross-species transferability was tested against tomato, pepper and potato DNA. DNA was isolated from young leaves using the method described by Doyle and Doyle [54]. PCR amplification was carried out in 20 μl reactions, each containing 10 ng genomic DNA, 10 nmol/L Tris-HCl (pH 8.3), 50 mmol/L KCl, 2.5 mmol/L MgCl2, 0.5 U Taq polymerase, 0.2 mmol/L dNTP, 200 nmol/L unlabeled reverse primer and 200 nmol/L IRD700-labelled forward primer. A touchdown PCR protocol was applied, consisting of a 94°C/5 min denaturation, 11 cycles of 94°C/30 s, 60°C/30 s decreasing by 0.5° per cycle, and 72°C/60 s, followed by 30 cycles of 94°C/30 s, 55°C/30 s and 72°C/60 s. The success of each amplification was monitored by analysis of the reaction product following 2% agarose gel electrophoresis, and successful amplicons were separated by denaturing 6% polyacrylamide gel electrophoresis on a LI-COR Gene ReadIR 4200 device, as described by Jackson and Matthews [55]. Determination of amplicon size was achieved by including an lRD700-labelled 50–350 bp ladder in each well. The data were collected by e-Seq software (DNA Sequencing and Analysis Software) v3.0.

### Data analysis

The polymorphism information content (PIC) of an SSR combines the number of alleles and their frequency distribution within a population [56]. For the present purposes, it was estimated as by Anderson et al. [57]. The SSR products were scored as band presence (1) and absence (0), thus generating a binary matrix. The binary data matrix was used to compute pair-wise similarity coefficients [58], and the similarity matrices obtained were utilized to construct a UPGMA-based dendrogram [58]. Principal co-ordinate analysis (PCO) was carried out to display the multi-dimensional relationship between accessions. All analyses were performed using the NYSYS software package v2.10 [60].

## Authors' contributions

SL and GLR planned and supervised the work. AS carried out SSR mining, primer design and amplification; LT and GLR provided plant materials; EP carried out the analysis of data. All the authors contributed to the final version of the manuscript.
